# Terroir Is the Main Driver of the Epiphytic Bacterial and Fungal Communities of Mango Carposphere in Reunion Island

**DOI:** 10.3389/fmicb.2020.619226

**Published:** 2021-01-20

**Authors:** Ahmed Taîbi, Ronan Rivallan, Véronique Broussolle, Dominique Pallet, Sylvie Lortal, Jean-Christophe Meile, Florentin Constancias

**Affiliations:** ^1^CIRAD, UMR Qualisud, Saint-Pierre, France; ^2^Qualisud, Univ Montpellier, Avignon Université, CIRAD, Institut Agro, Université de La Réunion, Montpellier, France; ^3^CIRAD, UMR AGAP, Montpellier, France; ^4^AGAP, Univ Montpellier, CIRAD, INRAE, Institut Agro, Montpellier, France; ^5^INRAE, Avignon Université, Sécurité et Qualité des Produits d’Origine Végétale, Avignon, France; ^6^CIRAD, UMR Qualisud, Montpellier, France; ^7^INRAE, Département Microbiologie et Chaine alimentaire, Jouy-en-Josas, France

**Keywords:** metabarcoding, epiphytic microbiota, mango (*Mangifera indica*), cultivar, biogeography, bacterial communities, fungal communities

## Abstract

The diversity of both bacterial and fungal communities associated with mango surface was explored using a metabarcoding approach targeting fungal ITS2 and bacterial 16S (V3-V4) genomic regions. Fruits were collected in Reunion Island from two different orchards according to a sampling method which allowed the effect of several pre-harvest factors such as geographical location (terroir), cultivars, fruit parts, tree position in the plot, fruit position on the tree (orientation and height), as well as the harvest date to be investigated. A total of 4,266,546 fungal and 2,049,919 bacterial reads were recovered then respectively assigned to 3,153 fungal and 24,087 to bacterial amplicon sequence variants (ASVs). Alpha and beta diversity, as well as differential abundance analyses revealed variations in both bacterial and fungal communities detected on mango surfaces depended upon the studied factor. Results indicated that *Burkholderiaceae* (58.8%), *Enterobacteriaceae* (5.2%), *Pseudomonadaceae* (4.8%), *Sphingomonadaceae* (4.1%), *Beijerinckiaceae* (3.5%), and *Microbacteriaceae* (3.1%) were the dominant bacterial families across all samples. The majority of fungal sequences were assigned to *Mycosphaerellaceae* (34.5%), *Cladosporiaceae* (23.21%), *Aureobasidiaceae* (13.09%), *Pleosporaceae* (6.92%), *Trichosphaeriaceae* (5.17%), and *Microstromatales_fam_Incertae_sedis* (4.67%). For each studied location, mango fruit from each cultivar shared a core microbiome, and fruits of the same cultivar harvested in two different locations shared about 80% fungal and bacterial family taxa. The various factors tested in this study affected bacterial and fungal taxa differently, suggesting that some taxa could act as geographical (terroir) markers and in some cases as cultivar fingerprints. The ranking of the factors investigated in the present study showed that in decreasing order of importance: the plot (terroir), cultivar, fruit parts, harvest date and the position of the fruits are respectively the most impacting factors of the microbial flora, when compared to the orientation and the fruit position (height) on the tree. Overall, these findings provided insights on both bacterial and fungal diversity associated with the mango surface, their patterns from intra-fruit scale to local scale and the potential parameters shaping the mango microbiota.

## Introduction

Fruits harbor on their surface a diversity of microorganisms (Leff and Fierer, 2013; Abdelfattah et al., 2016a), which can play a central role in fruit health, as it could be view as part of the solutions against some fruit diseases. Increasing studies highlighted the epiphytic microbial diversity (i.e., microbial communities associated with fruit surface) and the importance of the fruit microbiome (Janisiewicz et al., 2013; Pinto et al., 2014; Kecskeméti et al., 2016; Abdelfattah et al., 2018; Droby and Wisniewski, 2018). The microbial communities of fruit surfaces being an open ecosystem exposed to different biotic and abiotic factors (Barata et al., 2012; Ottesen et al., 2013), the fluctuations in microbial diversity are poorly understood probably because their study is challenging. Diverse factors are known to influence epiphytic microbial communities, such as the surrounding air (i.e., pollutants, treatments) (Joshi, 2008; Abdelfattah et al., 2019), soil richness (Berg and Smalla, 2009), terroir or producing region (Mezzasalma et al., 2018) as well as intrinsic factors (i.e., tree age, cultivar, stress, genetic variability, rootstocks/scion, diseases, fruit physiology and anatomy) (Leff and Fierer, 2013; Diskin et al., 2017; Liu et al., 2018; Marasco et al., 2018; Vepštaitė-Monstavičė et al., 2018).

Soil is the first reservoir of plant microbiomes (Barata et al., 2012; Bacon and White, 2016). The rhizosphere corresponds to the zone of soil surrounding a plant root that contains the highest microbial diversity, especially in bacteria, where microbes are at the shelter of the different abiotic factors (i.e., temperature, humidity, and UV radiation variations) affecting the aerial parts such as the leaf, flower, and fruit (Ottesen et al., 2013). Various studies showed the existence of a decreasing gradient of microbial richness and diversity between different parts of the plant located at an increasing distance from the soil (Ottesen et al., 2013; Abdelfattah et al., 2016b; Trivedi et al., 2020). Microorganisms that colonize the surface of the fruit can be transported from the soil to different organs of the plant (stem, leaves, flowers and fruit) by insect and animal species as well as raindrop splash and wind (Valero et al., 2007; Compant et al., 2011; Stefanini et al., 2012; Martins et al., 2013; Berg et al., 2014; Zarraonaindia et al., 2015; Mezzasalma et al., 2018).

Only few studies focusing on the carposphere microbiota have been published yet, mostly on grapes and apples (Setati et al., 2012; Bokulich et al., 2014; Taylor et al., 2014). The influence of geographical locations, farming practices, plant cultivars, harvest date and fruit parts was previously reported in separate studies, especially on grapes (Pretorius, 2000; Setati et al., 2012; Pinto et al., 2014, 2015; Wang et al., 2015; Zarraonaindia et al., 2015) and apples (Leff and Fierer, 2013; Abdelfattah et al., 2016a; Diskin et al., 2017; Liu et al., 2018; Vepštaitė-Monstavičė et al., 2018). These studies clearly showed that microbial communities composition differ strongly according to plant species (Lindow and Brandl, 2003; Hunter et al., 2010; Leff and Fierer, 2013; Vepštaitė-Monstavičė et al., 2018), and also to climatic conditions, ripening stage and the application of agrochemicals (Pretorius, 2000; Pinto et al., 2014, 2015; Liu et al., 2016). However, little is known on the potential influence of other factors and their relative contribution in shaping the carposphere microbiota.

Some previous studies used fruits from grocery stores, without taking into account the period between fruit harvest and analyses including human contamination by handling, storage and transport conditions that could have a significant impact of on microbial communities. Neglecting the period between harvest and sampling analysis therefore potentially leading to a misinterpretation of the results.

The domesticated mango *(Mangifera indica L.)* is native from the Indian subcontinent. The mango tree was introduced in many tropical and subtropical regions (Mukherjee, 1950, 1953) where it adapted to a wide variety of climates and soils, with preferred semi-arid areas, with alternating dry and wet periods. There are hundreds of mango cultivars harboring specific characteristics such as taste, flesh and peel color, size, shape, resistance to transport and storage, resistance to diseases and insects. Few studies conducted on mango-associated microbiomes focused on the biocontrol of mango diseases, such as anthracnose and stem-end rot diseases (Ortega-Morales et al., 2009; Bautista-Rosales et al., 2013; Rungjindamai, 2016; Diskin et al., 2017), but no study exploring the microbial diversity of mango fruit surface using metabarcoding approaches was reported so far.

In this context, the objectives of this present study were: (*i*) to provide an exhaustive characterization of both the fungal and bacterial communities associated with mango surface, (*ii*) to identify and (*iii*) rank the pre-harvest factors influencing bacterial and fungal communities.

In order to achieve those objectives, the sampling strategy of this present study was designed to include several levels of comparison. Thus, fruits were collected from two orchards to be representative of the plot, and several pre-harvest factors including the geographical location (plot), cultivars, within fruit parts, tree position in the plot, fruit position on the tree, as well as the harvest date. Mangoes were harvested in sterile conditions to avoid any contamination from human skin microbiome and molecular characterization of both bacterial and fungal communities using 16S V3-V4 and ITS2, respectively, were performed. The potential influence of the different factors was taken into account community analysis using both 16S V3-V4 and ITS2 datasets. By getting more insight into the mango epiphytic bacterial and fungal communities, we believe that post-harvest fruit handling and transformation steps as well as potential biocontrol strategies could be further developed.

## Materials and Methods

### Mango Sampling and Experimental Design

**FIGURE 1:**
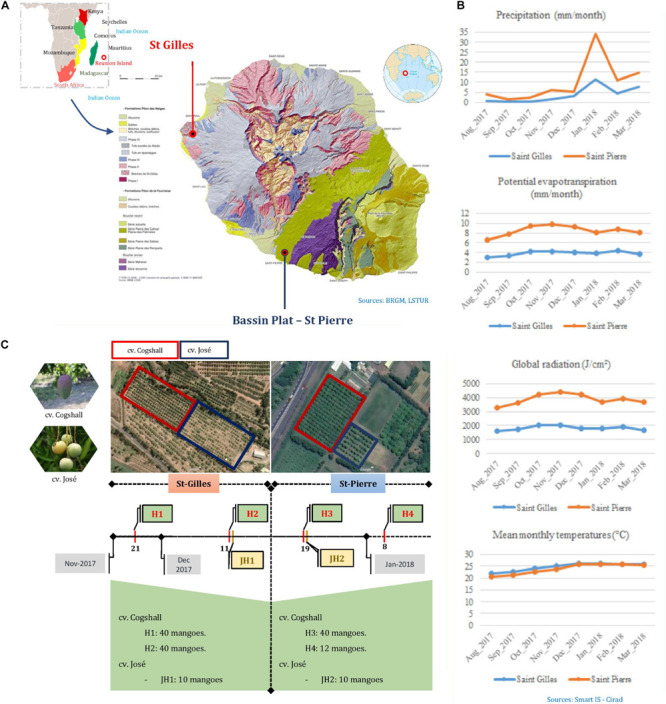
Synthetic diagram of the sampling protocol. **(A)** The geographic location of the two plots in Reunion; photos of the two mango cultivars and their location on the two geographic sites. **(B)** Pedoclimatic conditions of the sampling sites (Saint Gilles in blue and Saint Pierre in red). **(C)** The number of samples according to cultivars, terroir and date of harvest; H, “Harvest”; JH, “José Harvest.”

Fruits were harvested during the mango season peak between November 2017 and January 2018 in Reunion Island (Indian Ocean), in two different areas. St-Gilles (21°02′28.4″S 55°13′34.5″E, 49 meters of altitude) and Bassin Plat (21°19′22.6″S 55°29′17.2″E, 140 meters of altitude). Each area is characterized by specific microclimatological conditions. The following data were collected from the local weather stations: mean air temperatures in November 2017 and January 2018 were 25.5 and 24.95 C in St-Gilles and Bassin plat, respectively, sunshine 10 h/d, rainfall were 3.67 mm/month and 6.24 mm/month, respectively. The two selected plots were separated by a distance of 40 km. Both plots shared some characteristics; mango cultivars, sun exposure and management system. The last phytosanitary treatment (Product: Karate Zeon; Active ingredient: Lambda Cyhalothrin; Dose: 0.00125L/hL) was applied during the flowering period between August and September 2017 in St Pierre. Two mango cultivars were selected: Cogshall (‘American’ cultivar) and José (local cultivar). Two sampling harvests of cv. Cogshall were performed in each plantation plot [two harvest dates in St-Gilles (H1, H2) and two harvest dates in St-Pierre (H3, H4)], similarly one fruit batch of cv. José was harvested in each location (St-Gilles JH1 and St-Pierre JH2) as shown in Figure 1. In order to evaluate the cultivar and geographical impact on the composition of microbial communities, fruit samples were collected at the same stage of maturity (pre-climacteric state also named ‘Green mature’) according to their peel color, without any visible mechanical, insect damage or fungal diseases using sterilized gloves and placed in sterile bags. Sampled fruits were transported to the laboratory and analyzed within 2h after harvest from the two plantations. In total, 90 mangoes [80 fruits of cv. Cogshall (H1 and H2), and 10 fruits of cv. José (JH1)] were harvested in two sampling periods from St-Gilles, and similarly, 62 mangoes from St-Pierre (52 fruits of cv. Cogshall, H3 and H4, and 10 fruits of cv. José, JH2) (Figure 1). Fruits were collected from two orchards according to a sampling plan to be representative of the plot. Hence, the sampling design allowed to investigate the following pre-harvest factors: (i) the geographical location (St-Gilles and St-Pierre), (ii) the cultivar (cv. Cogshall and cv. José), (iii) the fruit parts (stem-end ‘SE’ and peel surface ‘PE’), (iv) the position of the tree in the plot (edge and center), (v) the position of the fruit on the tree (Height: <2.5 m vs. >2.5 m), (vi) orientation (East, North, and South), and (vii) the harvest dates (H1 and H2 in St-Gilles; H3 and H4 in St-Pierre).

### Microbial Sampling, DNA Extraction, PCR Amplification and Sequencing

Epiphytic microbial communities were sampled from each mango by swabbing the surface with a sterile single-use cotton swab previously wet with sterile buffered peptone water (Shen et al., 2018b,a), then placed in an individual tube filled with 1 ml qs of the same solution (Bodur and Cagri-Mehmetoglu, 2012) (pH 7.2 ± 0.2; peptone 10 g/L, sodium chloride 5 g/L, disodium phosphate 9 g/L, monopotassium phosphate 1.5 g/L; Merck, Darmstadt, Germany). For Fruit Part samples, the Stem-End surface (SE) and the rest of the total mango peel surface (PE) were swabbed from the same fruit and collected separately. The collected samples were stored at −20°C prior to DNA extraction. Total DNA was extracted from individual samples using Fast DNA Kits and the FastPrep^®^ homogenizer (MP Biomedicals, Inc., United States), according to the manufacturer’s instructions. DNA concentration was then quantified using Bisbenzimide Hoechst 33258 dye (Amersham Biosciences, Piscataway, NJ) (Sabnis, 2015), and normalized to 5 ng/μl^–1^. A two-step PCR strategy was performed combined with the dual-index paired-end sequencing approach described in Kozich et al. (2013). The 16S rRNA V3–V4 gene region and ITS2 region were amplified with specific archaeal/bacterial (341F: CCTACGGGNGGCWGCAG/785R: GACTACHVGGGTATCTAATCC) and fungal (ITS86F: GTGA ATCATCGAATCTTTGAA/ITS4R: TCCTCCGCTTATTGATA TGC) DNA primers (Op De Beeck et al., 2014; Thijs et al., 2017). PCR reactions were conducted in a total volume of 25 μl containing 12.5 μl of Phusion Flash High-Fidelity PCR Master Mix (Thermo Fisher Scientific, Lithuania), 0.625 μl of each primer (10 μM), and 5 ng template DNA. PCR reactions were incubated in a T100 thermal cycler (Bio-Rad, Hercules, CA, United States) for 3 min at 98°C followed by 25 cycles (for 16V3-V4) or 30 cycles (for ITS2) of [30 s at 95°C, 30 s at 50°C and 30 s at 72°C]. A final extension of 10 min at 72°C. Nuclease-free water replaced template DNA in negative controls. PCR products were visualized by agarose gel electrophoresis (1.5% in TAE buffer). Amplicon DNA yields from each PCR reaction were then quantified using Hoechst method. All PCR products were normalized to equimolar concentrations. Sequencing libraries for each sample were generated in accordance with the Illumina 16S rRNA metagenomic sequencing library preparation protocol (Kozich et al., 2013). Sequencing was performed on an Illumina MiSeq at The Regional Genotyping Platform of the UMR AGAP (Univ Montpellier, CIRAD, INRAE, Institut Agro, Montpellier, France) core facility.

### Bioinformatics

Illumina Miseq reads were processed similarly as previously described (Santillan et al., 2019). Briefly, Illumina adaptors and gene-specific primers were removed using Cutadapt (Martin, 2011). Sequences were then processed using the DADA2 pipeline (Callahan et al., 2016) which allows inference of exact amplicon sequence variants (ASVs). Bacterial (16S) reads were truncated after 260 and 230 nucleotides for forward and reverse reads, respectively. ITS2 sequences, reads were not truncated in order to allow the capture of size polymorphism of ITS sequences. Then, 16S and ITS2 reads with expected error rates higher than 2 and 4 for forward and reverse reads were removed. After filtering, error rate learning, ASV inference and denoising, reads were merged with a minimum overlap of 20 bp. Chimeric sequences were identified and removed. Taxonomy was assigned for 16S and ITS2 ASV using SILVA (v.132) (Glöckner et al., 2017) and UNITE (v. 1.3.3) (Abarenkov et al., 2010; Nilsson et al., 2013) databases, respectively.

### Statistics

For all studied factors, we tried to reduce the data sets to avoid a cumulative effect. For the terroir and cultivar factors, a reduced dataset was used carried out by considering only the data corresponding to the cv. Cogshall fully swabbed and not used in the “fruit parts” section. ASV tables were rarefied to an even depth of 2, 700 reads per sample for both 16S V3-V4 and ITS2 datasets allowing robust use of alpha and beta diversity metrics (Willis, 2019; Mbareche et al., 2020). Observed species (number of observed ASV) and Chao1 indices (richness + estimated number of unobserved ASV) were used to quantify the richness (Chao, 1987; Hughes et al., 2001). Diversity was quantified via Shannon (evenness of the species abundance distribution) and InvSimpson indices. The Kruskal–Wallis tests (KW) were used to search for significant differences in alpha-diversity among groups.

Bray–Curtis and Sørensen β-diversity dissimilarities were used in order to characterize community structure and composition, respectively (McArdle and Anderson, 2001). Ordination technics (PCoA and NMDS) as well as hierarchical clustering (with the ward.D2 method) were employed to depict community structure and composition differences between samples (Lema et al., 2019). PERMANOVA (Permutational multivariate analysis of variance), Analysis of similarities (ANOSIM) and multi-response permutation procedures (MRPP) were conducted to assess the source of variation of β-diversity measures (Anderson, 2017). Fungal and bacterial datasets were also used to generate differential abundance plots between categories using the DESeq2 (Anders and Huber, 2010) with alpha of 0.01 and fold change of 2 (log2 scale). Tanglegram analysis was conducted to compare bacterial and fungal dendrograms based on community similarities (Bray–Curtis distance). The two dendrograms with the same set of tip labels connected by lines, were plotted using the dendextend R-package (v. 1.13.4) (Galili, 2015). The phylogenetic analysis was conducted by extracting the tree from 16S dataset and visualized using the Interactive Tree of Life^1^ (Letunic and Bork, 2019). Statistical analyses were conducted using Rstudio [with the phyloseq (v 1.28.0)] (McMurdie and Holmes, 2013), microbiome (v. 1.6.0) (Lahti et al., 2017), Vegan (v. 2.5-6) (Oksanen et al., 2019), ggplot2 (v. 3.2.1) (Wickham, 2016), igraph (v. 1.2.4.1) (Csardi and Nepusz, 2006), DESeq2 (v. 1.24.0) (Love et al., 2014), pheatmap (v. 1.0.12) (Kolde, 2012), UpSetR (v. 1.4.0) (Lex et al., 2014) packages in R [version 3.6.1 (2019-07-05)].

### Results

A total number of 2,049,919 and 4,266,546 reads were processed for 16S V3-V4 and ITS2 datasets, respectively. DADA2 pipeline allowed to identify 24,087 and 3,153 bacterial and fungal ASVs, respectively after removing singletons, as well as ASV assigned to non-target plant sequences (*i.e.*, Alveolata, Metazoa, unknown phylum, Eukaryota and Chloroplast), represented 6 Phyla, 30 Classes, 90 Orders, 245 Families, 523 Genera, and 725 Species; while bacterial ASVs represented 24 Phyla, 45 Classes, 123 Orders, 301 Families, 893 Genera, and 566 Species.

**FIGURE 2:**
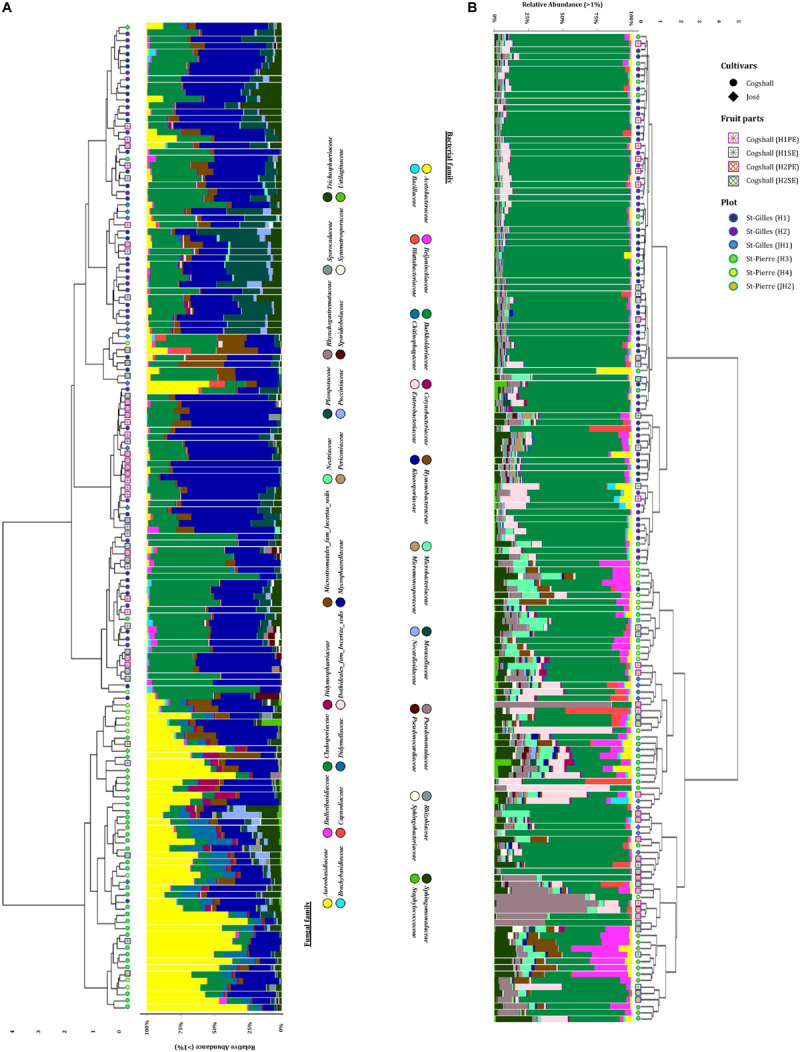
Hierarchical clustering of all samples based on community structure. Samples were hierarchically clustered based on their communities using the Bray-Curtis dissimilarity measure, which was visualized in the dendrogram. Adjacent to the branches of the dendrogram information on plot (St-Gilles: “H1/H2/JH1”; St-Pierre: “H3/H4/JH2”), cultivars (cv. Cogshall, cv. José) and fruit parts (PE: “H1/H2”; SE: “H1/H2”) is shown. The relative abundance of the 20 highest ranking family taxa is shown per sample in vertical stacked bar plots. **(A)** Fungal dendrogram based on community similarities (Bray–Curtis distance) derived from ITS2 sequences. **(B)** Bacterial dendrogram based on community similarities (Bray–Curtis distance) derived from 16S rRNA gene sequences.

**FIGURE 3:**
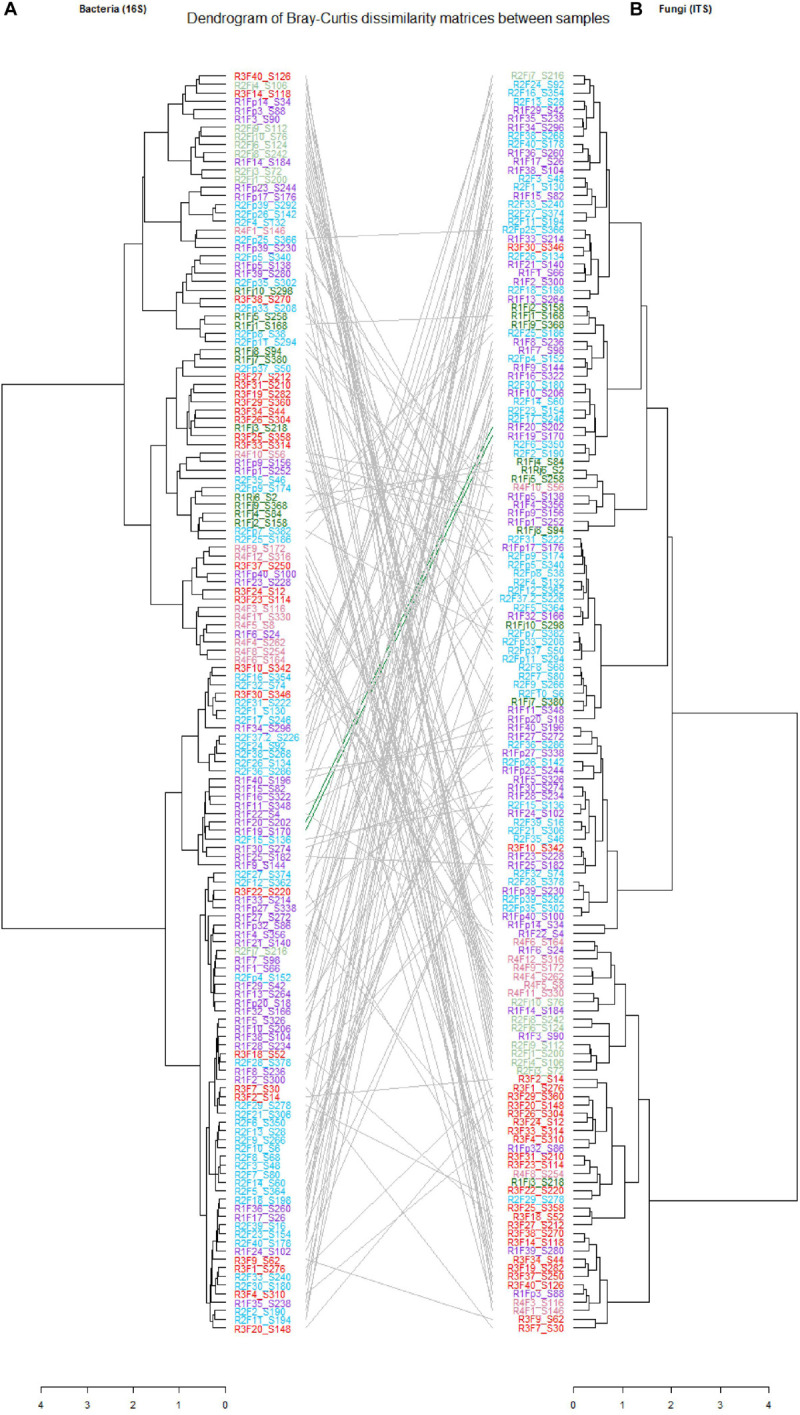
Tanglegrams showing concordance between bacterial and fungal dendrograms from mango fruit samples. The lines in the tanglegram connect the samples. Individual dendrograms were calculated using hierarchical clustering with the ward. D2 method, **(A)** Bacterial dendrogram based on community similarities (Bray–Curtis distance) derived from 16S rRNA gene sequences. **(B)** Fungal dendrogram based on community similarities (Bray–Curtis distance) derived from ITS2 sequences.

In this study, we characterized both bacterial and fungal communities from 152 mangoes to identify and rank the environmental parameters shaping the mango microbiota, including the main factors as terroirs, cultivars, fruits parts and harvest date. In general, we found that the factors studied had a differential influence on the distribution and diversity of the bacterial and fungal communities, especially terroir (Figure 2). Hierarchical clustering of all samples based on both bacterial and fungal community compositions shows that geographical location had more influence on fungal (relative to bacterial) community structures. In addition, tanglegram between bacterial and fungal dendrograms show that the fungal and bacterial structures were differentially affected by pre-harvest factors (Figure 3 and Supplementary Data 8). For instance, Aureobasidium was most abundant in St-Pierre and *Cladosporiaceae* was most abundant in St-Gilles, which seems to act as geographical (terroir) markers (Figure 2). In the following sections will be presented and discussed the data related to factors for which a significant impact on microbial communities was observed.

### Mango Fruit Geographical Origin and Microbial Composition

**FIGURE 4:**
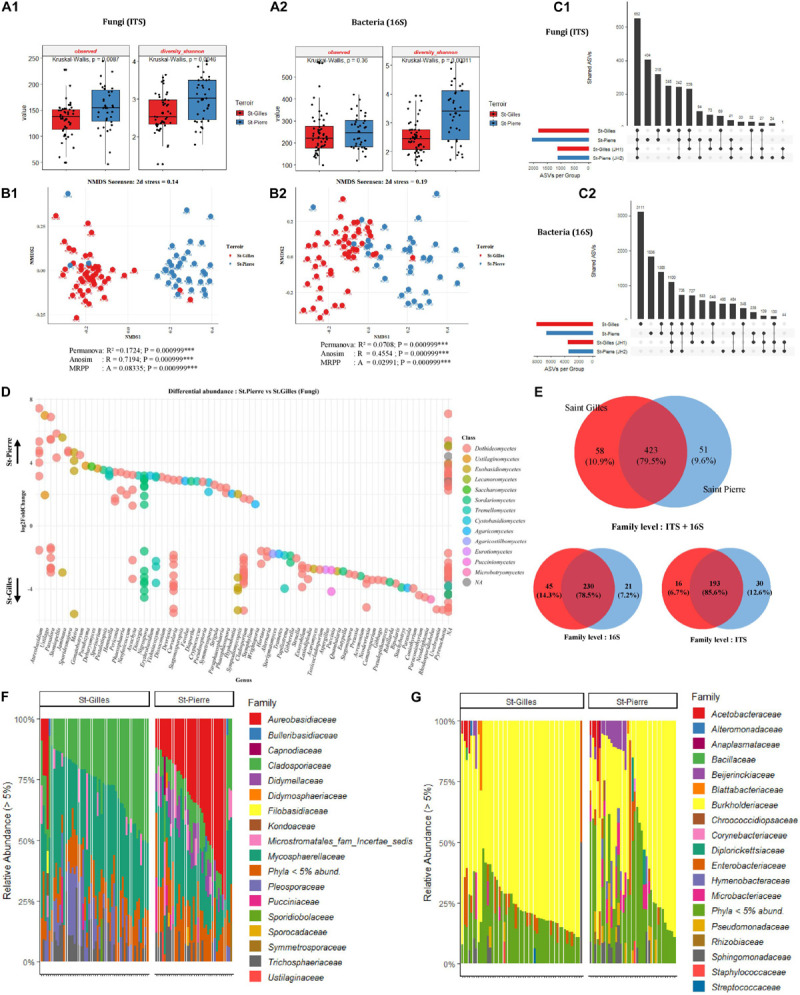
Comparison of alpha, beta diversity and community composition between cv. Cogshall samples collected from St-Gilles and St-Pierre. **(A)** Boxplots of different diversity indices (alpha, InvSimpson, Shannon, Observed, and Chao1) for Fungal **(A:1)** and Bacterial **(A:2)** ASVs. **(B)** Non-metric multidimensional scaling (NMDS) plot based on the distance matrix of Sørensen dissimilarity of microbial communities between samples for Fungal **(B:1)** and Bacterial **(B:2)** ASVs. The asterisk represents the statistical difference between categories by PERMANOVA, ANOSIM and MRPP. The number of stars account for *p*-value range: (***) *P* ≤ 0.001. A rule of thumb, stress levels less than 0.2 indicate a good representation of the data in a reduced number of dimensions. **(C)** UpSetR plot of unique and shared fungal **(C:1)** and bacterial **(C:2)** ASVs among samples harvested in St-Gilles and St-Pierre. **(D)** Differential abundance analysis (DESeq2) expressed as Log2FC comparison of St-Pierre vs. St-Gilles samples, Positive Log2FC represents phyla more abundant in St-Pierre samples indicated by up-arrow. Negative Log2FC represents phyla more abundant in St-Gilles samples indicated by down-arrow and each point represents an individual ASV. ASVs are organized on the *x*-axis according to Genus classification level and colored in the legend according to their Class level. Only ASVs with significant differences (*P*-adjusted < 0.01) in log2 fold change are depicted. **(E)** Venn diagrams showing the number of unique and shared fungal (ITS) and bacterial (16S) families between samples. **(F,G)** Relative abundance at the family level in cv. Cogshall collected from Saint Gilles and Saint Pierre; (F) Fungal and (G) bacterial ASVs.

Analysis of the bacterial and fungal community composition according to the terroir was performed. To this end, the diversity and richness indices of all cv. Cogshall samples from the two regions were measured to describe the complexity of each sample. The highest number of fungal ASVs was detected in cv. Cogshall mango sampled in St-Pierre with a significant difference between plots (Observed [KW *p*-value = 0.0087]), while the same trend was measured for bacterial communities with no significant difference (Observed [KW *p*-value = 0.36]) (Figure 4A2). Fungal communities were significantly more diverse in St-Pierre as compared to St-Gilles (Shannon [KW *p*-value = 0.004641]). Similarly, the same trend was measured for bacterial communities (Shannon [KW *p*-value = 0.00011]) (see Figures 4A1,A2 and Supplementary Data 1, 2).

For both bacterial and fungal datasets, differences in community composition between samples are depicted by NMDS ordination based on Sørensen dissimilarity. Samples clearly cluster according to their geographical origin which is confirmed by statistical tests based on fungal communities (PERMANOVA pseudo-F = 16.9; *R*^2^ = 0.17, *p* < 0.001; ANOSIM: *R* = 0.72; *P* < 0.001; MRPP: *P* < 0.001, see Figures 4B:1,B:2). Nevertheless, samples from St-Gilles and St-Pierre were less clearly separated in the plot based on bacterial communities (PERMANOVA pseudo-F = 6.4, *R*^2^ = 0.071, *p* < 0.001; ANOSIM: *R* = 0.46; *P* < 0.001; MRPP: *P* < 0.001) (see Figures 4B1,B2 and Supplementary Data 3).

Six fungal phyla were dominant in samples harvested in St-Gilles, *Ascomycota* (53,13%), *Basidiomycota* (15.07%), *Mucoromycota* (8.22%), *Mortierellomycota* (8.12%), *Chytri- diomycota* (7.73%) and *Blastocladiomycota* (7.73%) (Figure 4F). A similar trend was observed in samples harvested in St-Pierre, were the four most abundant fungal phyla detected were: *Ascomycota* (87.15%), *Basidiomycota* (12.85%), *Chytridiomycota* (0.002%) and *Blastocladiomycota* (0.0001%). *Mucoromycota* (8.22%) and *Mortierellomycota* (8.12%) were only detected in St-Gilles samples (Supplementary Data 5).

The 9 shared fungal families were *Mycosphaerellaceae* (36.72 and 22.38%), *Cladosporiaceae* (27.56 and 11.8%), *Aureoba- sidiaceae* (2.95 and 32.12%), *Microstromatales_fam_Incertae_ sedis* (3.71 and 5.08%), *Trichosphaeriaceae* (7.7 and 5.92%), *Didymellaceae* (0.65 and 7.2%), *Pleosporaceae* (11.19 and 2.39%), *Pucciniaceae* (2.06 and 1.8%), *Didymosphaeriaceae* (0.45 and 1.57%) families were the most abundant taxa shared between samples from the two geographical locations in St-Gilles and St-Pierre respectively (Figure 4F and Supplementary Data 5, 6).

Four dominant bacterial phyla (*Proteobacteria, Actinobacteria, Bacteroidetes, and Firmicutes)* were detected in all samples with a similar relative abundance pattern in both locations. *Proteobacteria* were detected as the most abundant phylum comprising 88.25 and 78.98% in St-Gilles and St-Pierre samples, respectively. The second most abundant bacterial phylum was *Actinobacteria* which was found with a similar relative abundance in St-Pierre (9.90%) and St-Gilles (5.5%) samples. The third most abundant phylum was *Bacteroidetes*, with relative abundance of 5.5 and 2.19% in St-Pierre and St-Gilles samples, respectively. *Firmicutes* represented about 2.88% in St-Pierre and 2.31% in St-Gilles of bacterial phyla (Supplementary Data 6). Different rare phylum taxa were detected with relative abundances of less than 1% (17 taxa at St-Pierre and 20 taxa at St-Gilles). Ten bacterial families were shared by samples from both locations with different proportions [e.g., *Burkholderiaceae* (71.74 and 51.51%), *Enterobacteriaceae* (4.65 and 4.96%), *Pseudomonadaceae* (1 and 3.75%), *Sphingomonadaceae* (3.26 and 4.72%), *Microbacteriaceae* (1.55 and 4.56%), *Beijerinckiaceae* (1.38 and 4.90%), *Hymenobacteraceae* (0.25 and 2.64%), *Rhizobiaceae* (1.66 and 1.42%), *Acetobacteraceae* (1.62 and 2.52%), and *Blattabacteriaceae* (0.94 and 1.14%)] in St-Gilles and St-Pierre, respectively (Figure 4G).

The Core microbiome of “cv. Cogshall” collected from St-Gilles and St-Pierre consisted of 230 bacterial (78.5%) and 193 fungal (85.6%) families, which represented respectively 79.5% of the total family taxa (532) detected in Cogshall cultivar samples. In other words, 45 bacterial (14.3%) and 16 fungal (6.7%) families were detected exclusively at St-Gilles, while 21 bacterial (7.2%) and 30 fungal (12.6%) families were exclusively detected in St-Pierre (Figure 4E). About 1,817 fungal and 8,085 bacterial ASVs in St-Gilles and 2,053 Fungal and 6,646 bacterial ASVs in St Pierre were detected on cv. Cogshall samples. About 248 fungal and 3,111 bacterial ASVs were exclusively detected in St-Gilles and 404 fungal and 1,836 bacterial ASVs in St-Pierre. 3948 bacterial ASVs and 1441 fungal ASVs were shared by the two terroirs (Figure 4C), which represent about 49% of the total ASVs detected on cv. Cogshall mangoes analyzed. In addition, about 1,125 fungal and 3,510 bacterial ASVs in St-Gilles and 1,113 fungal and 3,466 bacterial ASVs in St Pierre were detected on cv. José cultivars.

Differentially abundant bacterial taxa were identified using DESeq2 method and plotted in Figure 4D. In total, 89 bacterial ASVs were more abundant in St-Pierre than in St-Gilles. Most of those taxa belong to one of the 3 bacterial families: *Beijerinckiaceae*, *Burkholderiaceae* and *Sphingomonadaceae*. For fungal ASVs, the differential abundance analysis identified 95 fungal ASV that were more abundant in St-Pierre and 149 ASVs that were more abundant in St-Gilles. Most of those fungal taxa belong to one of the 5 following families: *Cladosporiaceae*, *Didymellaceae*, *Mycosphaerellaceae*, *Pleosporaceae*, *Trichosphaeriaceae*. At least 7 fungal genera including (but not limited to) *Stomiopeltis*, *Cryptococcus*, *Stagonosporopsis*, *Phaeophleospora*, *Sporidesmajora*, *Phaeosphaeria*, *Paraphaeosphaeria*, were significantly more abundant (*p*-adjusted < 0,001) in samples from St-Pierre, while 7 other fungal genera, *Neodevriesia*, *Stachybotrys*, *Rhodosporidiobolus*, *Gibberella*, *Paraconiothyrium*, *Alternaria*, *Exserohilum* genera were the most-significantly differentially abundant taxa associated with samples from St-Gilles (Figure 4D and Supplementary Data 4).

### Mango Cultivars Shaping the Microbial Diversity and Distribution

**FIGURE 5:**
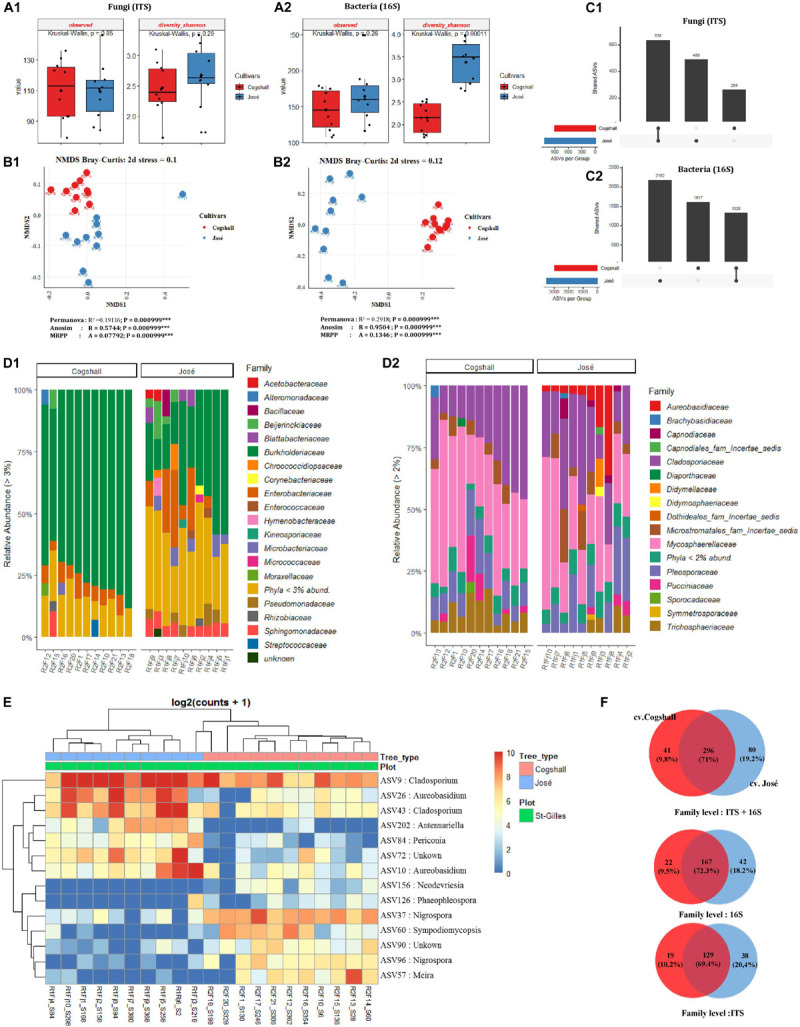
Alpha, beta diversity and community composition of cv. Cogshall and cv. José samples collected from St-Gilles. **(A)** Boxplots of different alpha diversity indices; diversity Shannon, Observed species for Fungal **(A:1)** and Bacterial ASVs **(A:2)**. Boxplots showed there was significant difference between cultivars samples. **(B)** Non-metric multidimensional scaling (NMDS) plot based on Bray-Curtis dissimilarity of the microbial community between samples, **(B:1)** Fungal and **(B:2)** Bacterial ASVs. The asterisk represents that there is significant difference between categories by PERMANOVA, ANOSIM and MRPP. The stars denote significance *p*-value: (ns) indicate *P* > 0.05; (^∗^) *P* ≤ 0.05; (^∗∗^) *P* ≤ 0.01; (^∗∗∗^) *P* ≤ 0.001. A rule of thumb, stress levels less than 0.2 indicate a good representation of the data in reduced dimensions. **(C)** UpSetR plot of unique and shared fungal **(C:1)** and bacterial ASV **(C:2)** among cultivars samples harvested in St-Gilles. **(D)** Relative abundance at the Family level in cv. Cogshall collected from Saint Gilles and Saint Pierre; **(D:1)** Bacterial and **(D:2)** Fungal ASV. **(E)** Heatmap of comparison of differential abundance analysis (DESeq2) of fungal ASVs between cultivars samples harvested in St-Gilles. Tree type and plot information are shown with colored bars at the top of the heatmap. Only ASVs with significant differences (*P*-adjusted < 0.01) in log2 fold change are depicted. **(F)** Venn diagrams showing the number of unique and shared fungal (ITS) and bacterial (16S) families between samples.

The influence of cultivars on microbial diversity was investigated by analyzing fruit samples from two cultivars cv. José (*n* = 10) and cv. Cogshall (*n* = 10) harvested in Saint Gilles. Regarding the fungal communities, data indicated that (alpha) diversity indices were higher in cv. Cogshall compared to cv. José samples (Figure 5A1 and Supplementary Data 1, 2), but the difference was not significant (Observed [KW *p*-value = 0.85]; Shannon [KW *p*-value = 0.29]). On the contrary, prokaryotic diversity (as shown by InvSimpson and Shannon indices) were higher in cv. José than in cv. Cogshall samples [Shannon (KW *p*-value = 0.0001]) (Supplementary Data 1, 2). Observed diversity index did not significantly differ between samples belonging to the two cultivars (Observed [KW *p*-value = 0.26]), indicating that diversity between samples belonging to the two cultivars are better related to Shannon index. Beta diversity indices based on both fungal and bacterial ASVs showed that samples belonging to the same cultivar are clustered together, which separate cv. Cogshall samples from cv. José samples, revealing different community composition according to the mango cultivar considered [Permanova (*P* < 0.001); Anosim (*P* < 0.001); MRPP (*P* < 0.001)] (Supplementary Data 3).

The 2 most abundant fungal phyla in all samples were Ascomycota (87.17 and 88.25%) and Basidiomycota (12.83 and 11.75%, on cv. Cogshall and cv. José samples respectively). The 4 most abundant bacterial phyla detected on both cultivar samples, Proteobacteria (90.78 and 69.67%), Actinobacteria (4.08 and 12.98%), Bacteroidetes (0.92 and 5.79%), Firmicutes (2.81 and 6.42%) as well as 14 rare phyla (<1%). Entotheonellaeota (4.09E-03%) and Tenericutes (9.13E-04%) were exclusively detected on cv. Cogshall fruits, while Nitrospirae (2.54E-01%), Spirochetes (1.22E-02%) and Epsilonbacteraeota (6.12E-03%) were only detected in cv. José (Supplementary Data 6). All samples harbored 15 dominant bacterial families such as *Burkholderiaceae* (75.07 and 38.3% on cv. Cogshall and cv. José samples, respectively), *Pseudomonadaceae* (0.82 and 3.5%), *Enterobacteriaceae* (6.13 and 10.1%), *Sphingomonadaceae* (1.81 and 5.4%), *Microbacteriaceae* (1.23 and 3.9%), *Beijerinckiaceae* (1.18 and 3.5%), *Rhizobiaceae* (1.68 and 1.6%), *Acetobacteraceae* (1.24 and 1.9%), *Blattabacteriaceae* (0.11 and 1.7%), *Hymenobacteraceae* (0.12 and 1.5%), *Bacillaceae* (0.44 and 1.8%), *Staphylococcaceae* (0.69 and 1.3%), *Corynebacteriaceae* (0.64 and 1.2%), *Chroococcidiopsaceae* (0.32 and 1.6%), *Micrococcaceae* (0.22 and 1.1%) (Figure 5D1 and Supplementary Data 6). Among other taxa which represented less than 1% of the total bacterial relative abundance, 175 and 195 other rare family taxa were detected on cv. Cogshall and cv. José, respectively. Similarly, the 9 most abundant fungal families, *Mycosphaerellaceae* (39.48 and 31.3%), *Cladosporiaceae* (25.4 and 25.1%), *Pleosporaceae* (11.5 and 13.08%), *Trichosphaeriaceae* (9.01 and 3.82%), *Aureobasidiaceae* (0.56 and 7.77%), *Microstromatales_fam_Incertae_sedis* (4.31 and 7.16%), *Pucciniaceae* (4.09 and 1.6%), *Didymellaceae* (0.38 and 1.42%), *Capnodiaceae* (0.11 and 2.15%) were detected respectively on both cv. Cogshall and cv. José samples (Figure 5D2 and Supplementary Data 5). Other rare taxa which represent less than 1% of the total fungal relative abundance were detected among which 139 and 158 rare families on cv. Cogshall and cv. José, respectively.

The two mango cultivars shared 167 bacterial (72.3%) and 129 fungal (69.4%) families at their surface, which represented 71% of all microbial families detected on the two cultivars harvested in St-Gilles. The seven dominant bacterial species shared by the two mango cultivars were *Passalora fulva* (60.18 and 36.7%), *Exserohilum rostratum* (8.89 and 7.65%), *Cladosporium sphaerospermum* (5.23 and 4.39%), *Cladosporium dominicanum* (4.83 and 16.5%), *Jaminaea angkorensis* (2.89 and 8.5%), *Curvularia hawaiiensis* (2.66 and 3.23%), *Aureobasidium pullulans* (0.46% in cv. Cogshall and 6.76% in cv. José, respectively). The bacterial species exclusively detected in cv. Cogshall samples were *Pelomonas saccharophila* (12.83%), *Pantoea eucalypti* (10.16%), *Massilia consociata* (5.41%), *Sphingomonas yunnanensis* (3.12%), *Massilia arvi* (2.68%) and *Corynebacterium accolens* (2.19%) The following species *Erwinia typographi* (4.84%), *Acetobacter pasteurianus* (4.21%), *Erwinia amylovora* (3.26%), *Kosakonia cowanii* (6.67%), *Sphingobium abikonense* (1.04%) (Supplementary Data 6). In cv. José, the 5 specific bacterial species detected were *Massilia oculi* (8.04%), *Morganella morganii* (6.76%), *Sphingomonas yunnanensis* (5.27%), *Methylobacterium aerolatum* (3.95%), and *Massilia arvi* (3.88%).

The two mango cultivars shared about 1,328 bacterial ASV and 636 fungal ASV. About 1,617 bacterial and 264 fungal ASVs were detected only in cv. Cogshall, while 2,182 bacterial and 489 fungal ASVs were detected only in cv. José (Figure 5C). Differential abundance analysis identified 7 ASVs that were highly abundant in cv. José while 7 ASVs that were highly abundant in cv. Cogshall (Figure 5E). The following species; *Antennariella placitae*, *Aureobasidium pullulans*, *Cladosporium sphaerospermum*, *Periconia digitata*, *Cladosporium dominicanum* had the highest abundance associated with cv. José samples, while *Nigrospora oryzae*, *Sympodiomycopsis paphiopedili*, *Neodevriesia pakbiae*, *Meira argovae* were significantly more abundant (*p*-value adjusted < 0.005) in cv. Cogshall samples (Supplementary Data 4).

### Distribution of Bacterial and Fungal Communities at the Fruit Scale (Peel Surface and Stem-End)

**FIGURE 6:**
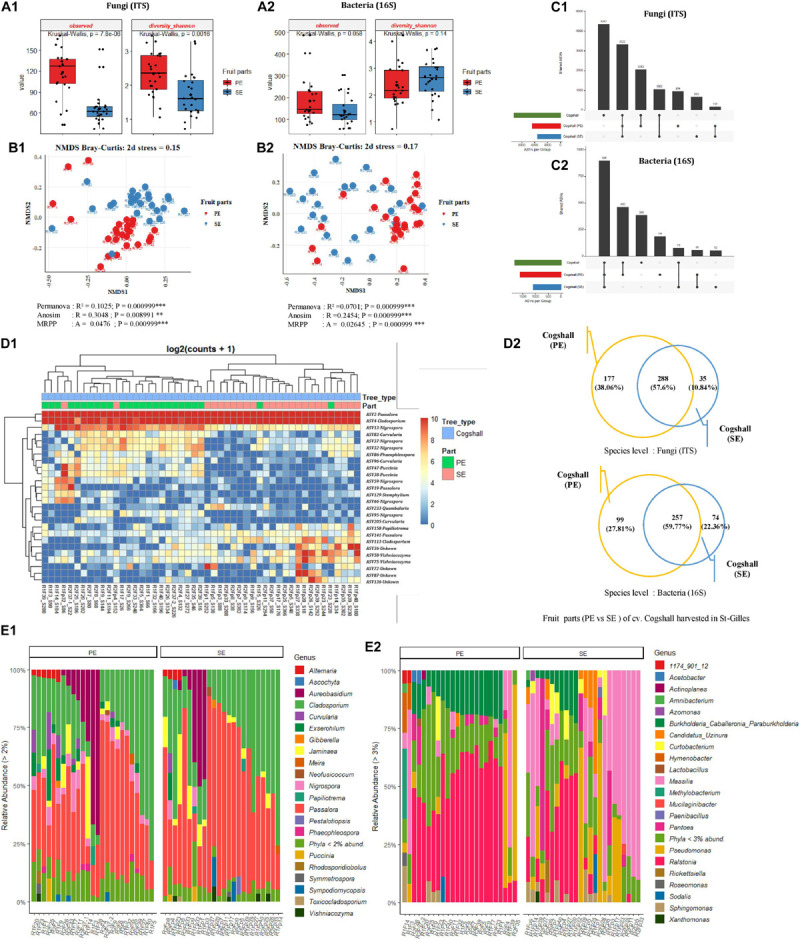
Comparison of alpha, beta diversity and community composition between stem-end (SE) and peel (PE) tissues from cv. Cogshall samples collected from St-Gilles. **(A)** Boxplots of different alpha diversity indices; Observed species and diversity Shannon for Fungal **(A:1)** and Bacterial ASVs **(A:2)**. Boxplots showed there was significant difference between samples from cv. Cogshall fruit parts. **(B)** Non-metric multidimensional scaling (NMDS) plot based on bray-Curtis dissimilarity of the microbial community between samples, **(B:1)** Fungal and **(B:2)** Bacterial ASVs. The asterisk represents a significant difference between categories by PERMANOVA, ANOSIM and MRPP. The stars denote significance of the *p*-value: (ns) indicate *P* > 0.05; (*) *P* ≤ 0.05; (**) *P* ≤ 0.01; (***) *P* ≤ 0.001. A rule of thumb, stress levels less than 0.2 indicate a good representation of the data in reduced dimensions. **(C)** UpSetR plot of unique and shared fungal **(C:1)** and bacterial ASV **(C:2)** among fruit parts zones. **(D:1)** Heatmap of comparison of differential abundance analysis (DESeq2) of fungal ASVs between fruit parts samples from cv. Cogshall harvested in St-Gilles. Tree type and fruit parts information are shown with colored bars at the top of the heatmap. **(D:2)** Venn diagrams showing the number of unique and shared fungal (ITS) and bacterial (16S) species between PE (peel surface) and SE (stem-end) samples. **(E)** Relative abundance at the Genus level in cv. Cogshall collected from Saint Gilles and Saint Pierre; **(E:1)** Fungal and **(E:2)** bacterial ASV.

For a subset of mango fruit harvested in St-Gilles plot, two different areas of the fruit (cv. Cogshall) were swabbed, the mango peel (PE, *n* = 24) and the stem-end (SE, *n* = 24) for microbial community analyses. The diversity and richness indices of all samples from the two different fruit parts were determined. The highest number of fungal ASVs was detected in mango peel (PE) compared to the stem-end (SE) part with significant differences (Observed [KW *p*-value = 7.8e-06]). A similar trend was observed regarding bacterial ASVs with no significant differences (Observed [KW *p*-value = 0,058]) (Figure 6A and Supplementary Data 1, 2). Fungal diversity was higher in PE zone compared to SE samples [Shannon (KW *p*-value = 0.002)] while Bacterial diversity showed an opposite trend [Shannon (KW *p*-value = 0.14)] (Figure 6A and Supplementary Data 1, 2). Beta diversity analysis (based on Bray-Curtis metric) showed revealing different microbial composition according to mango fruit parts. The presence of clusters separating PE samples from SE samples. Based on fungal ASVs analysis, PE and SE form clusters, indicating differences in the composition of fungal communities [PERMANOVA pseudo-F = 5.4, *R*^2^ = 0.10; ANOSIM: *R* = 0.31, *p* < 0.01; MRPP: *p* < 0,001]. A similar trend was observed with bacterial ASVs athough SE samples appeared less clustered than PE ones [PERMANOVA pseudo-F = 3.5, *R*^2^ = 0.07 *p* < 0.001; ANOSIM: *R* = 0.25; *p* < 0.001; MRPP: *p* < 0.001] (Figure 6B and Supplementary Data 3).

The 10 most abundant of bacterial genera were dominated by *Ralstonia* on both PE and SE samples (42.7 and 16.18%, respectively), followed by *Burkholderia* (13.17 and 4.6%), *Pseudomonas* (7.79 and 12.53%), *Massilia* (8.54 and 34.48%), *Pantoea* (2.68 and 6.71%), *Sphingomonas* (2.3 and 3.1%) *Methylobacterium* (1.81 and 0.69%), *Hymenobacter* (1.38 and 0.59%), *Curtobacterium* (1.44 and 3.81%) and *Candidatus_Uzinura* (0.58 and 4.24%) (Figure 6E2). The dominant sequences attributed to the 19 bacterial species found in the samples (with relative abundance of above 1%) were *Methylobacterium aerolatum* (5.76 and 4.46% in PE and SE samples respectively), *Massilia arvi* (8.24 and 19.17%), *Pelomonas saccharophila* (12.57 and 4.25%), *Sphingomonas yunnanensis* (4.07 and 5.43%), *Massilia consociata* (4.56 and 8.91%), *Pantoea eucalypti* (4.76 and 1.96%), *Pantoea ananatis* (0.42 and 2.1%), *Acetobacter pasteurianus* (5.43 and 0.14%), *Kosakonia cowanii* (3.05 and 0.08%), *Massilia niastensis* (1.14 and 8.01%), *Enhydrobacter aerosaccus* (1.31 and 0.79%), *Roseomonas ludipueritiae* (1.5 and 1.27%), *Erwinia typographi* (1.87 and 0.23%), *Sphingomonas desiccabilis* (0.53 and 2.87%), *Xanthomonas cynarae* (0.47 and 2.64%), *Amnibacterium_kyonggiense* (0.65 and 1.22%), *Curtobacterium flaccumfaciens* (0.14 and 1%), *Curtobacterium luteum* (1.54 and 2.24%), *Massilia kyonggiensis* (0.43 and 1.03%).

The 10 most abundant genera were dominated by *Passalora* (with respectively 37.03 and 47.33% in PE and SE samples), followed by *Cladosporium* (27.33 and 31.01%), *Aureobasidium* (9.45 and 6.62%), *Nigrospora* (4.72 and 0.88%), *Jaminaea* (3.78 and 4.26%), *Exserohilum* (3.28 and 0.66%), *Curvularia* (2.7 and 0.54%), *Alternaria* (1.74 and 1.04%), *Sympodiomycopsis* (0.79 and 1.21%) and *Vishniacozyma* (0.47 and 1.13%).

The 11 dominant sequences attributed to fungal species (with relative abundance > 1%) were *Passalora fulva* (53.17 and 66.08% in PE and SE respectively), *Jaminaea angkorensis* (3.82 and 5.2%), *Cladosporium sphaerospermum* (6.76 and 4.75%), *Cladosporium dominicanum* (4.37 and 4.82%), *Aureobasidium namibiae* (5.85 and 2.65%), *Aureobasidium pullulans* (2.11 and 3.08%), *Sympodiomycopsis paphiopedili* (0.72 and 1.23%), *Aureobasidium thailandense* (3.19 and 1.75%), *Vishniacozyma taibaiensis* (0.46 and 1.36%), *Exserohilum rostratum* (4.82 and 0.91%), and *Curvularia hawaiiensis* (1.85 and 0.39%) (Figure 6E1).

Overall, cv. Cogshall fruit parts (PE and SE) shared 288 fungal species (57.6%) and 257 bacterial species (59.7%), which represented respectively 58.6% of the total microbial species detected on the two fruit parts of cv. Cogshall mangoes harvested in St-Gilles (Figure 6D2). The two parts of the fruit share about 56 bacterial and 165 fungal ASVs, however, 184 bacterial and 954 fungal ASVs were exclusively detected in peel surface (PE), while 52 bacterial and 662 fungal ASVs only exclusively detected in stem-end (SE) as shown in Figure 6C. The comparison of differential abundance of fungal ASVs between stem-end (SE) and peel surface (PE) of cv. Cogshall samples harvested in St-Gilles identified 12 ASVs belonging to the 5 genera are (*Quambalaria*, *Vishniacozyma*, *Cladosporium*, *Papiliotrema*, and *Passalora)* that were highly abundant in Stem-end (SE). 14 ASVs, the majority of which belong 3 genera (*Nigrospora*, *Curvularia*, and *Puccinia*) were highly abundant in peel surface (PE) (Figure 6D1 and Supplementary Data 4).

### Ranking/Summary of the Factors Investigated in the Study

Our sampling allowed to investigate and rank the influence of seven factors on the composition of the microbiota associated with mango fruit surface. In this part, we will summarize and rank the most influencing factors to the least important factors in terms of microbial alpha and beta diversities. Alpha diversity data indicated that the terroir (plot) and the fruit parts had the most significant impact on mango fungal communities compared to other factors (particularly when compared to cultivars, fruit orientation and height as well as tree position and harvest date). However, regarding bacterial populations, the cultivar was the most impacting factor followed by the terroir (plot), the tree position, the fruit height, the fruit parts, the orientation and the harvest date. Overall, the terroir (plot) and fruit parts showed the highest effect on fungal diversity, while, the cultivar and the terroir (plot) strongly influenced the bacterial diversity while the tree position and harvest date impacted the bacterial richness (Supplementary Data 2).

Beta diversity analysis showed that bacterial and fungal diversity were differentially impacted (Supplementary Data 3). In general, all factors influenced both fungal and bacterial composition with different significance values except for fruit position on the tree [‘height’ (<2.5 m vs. >2.5 m)]. The terroir (plot), the cultivar, the fruit part, and the harvest date are the most influencing factors on both alpha and beta diversities based on the analysis of bacterial and fungal DNA sequences.

**FIGURE 7:**
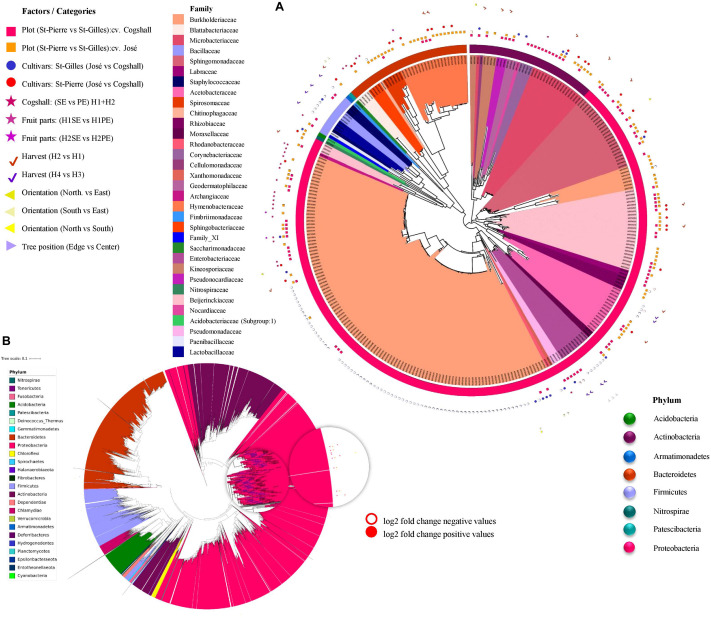
Differential abundance analysis (DESeq2) applied to different factors, including plot, cultivar, harvest date, fruit parts, orientation and tree position. Symbols aligned with bacterial ASVs impacted by factors in the phylogenetic tree. Each leaf on a phylogenetic tree represents a single ASV. A total 303 impacted ASVs belonging to 8 out of 25 phyla in bacterial dataset **(A)**. **(B)** Representation of the global bacterial ASV in filtrated dataset (24390 ASV). The log2(fold-change) is the log-ratio of an ASV’s differential abundance values in two different conditions, empty-shapes represents negative value of log2 fold-change and filled-shapes represents positive value of log2 fold-change.

Differentia abundance analysis (using DESeq2) was applied to each factor and the results are presented in Figure 7 (and Supplementary Data 7). Overall, 135 bacterial and 256 fungal ASVs (which represent 1.44% of total fungal and bacterial ASVs) were identified to be impacted by at least one of the factors studied. A given factor can influence the abundance of one or more ASVs, and several factors influenced the abundance of an ASV as shown in Figure 7.

Regarding bacterial ASVs, the terroir (plot) significantly affected 81 ASV, followed by the cultivar (47 ASV), the Harvest date ‘H1/H2’ (35 ASV), the Fruit parts (4 ASV), and the orientation (1 ASV) (Figure 7).

Regarding fungal ASV, DESeq2 results indicate that 188 ASV were impacted by only one factor presented in Supplementary Data 4 and 69 ASV were influenced by more than one factor. The terroir (plot) affected 244 fungal ASV, followed by the cultivar (14 ASV), the harvest dates ‘H1/H2’ (11 ASV)/‘H3/H4’ (30 ASV), and the Fruit parts (26 ASV) (Supplementary Data 4).

Overall, the terroir (plot) affected 325 fungal and bacterial ASVs, followed by the harvest dates (76 ASVs), the cultivars (61 ASVs), and the fruit parts (30 ASVs).

## Discussion

An increasing number of studies explored the microbiota of fruits and vegetables (Barata et al., 2012; Pinto et al., 2014; Abdelfattah et al., 2015, 2016a,b; Droby and Wisniewski, 2018; Hassan et al., 2018; Liu et al., 2018). Different factors may influence the distribution and composition of the microbial communities associated with the surface of the mango fruit. However, these factors are often interrelated, making it challenging to establish major and specific factors responsible in shaping bacterial and fungal communities (Leff and Fierer, 2013). Minor factors can have a cumulative effect and then significantly contribute to shaping the structure of the microbial communities. In the present study, our sampling strategy was design to gain insights on the factors shaping the mango fruit microbiota. Therefore, different modalities of terroir (St-Gilles vs. St-Pierre), tree position in an orchard (Edge vs. Center), position of the fruit on the tree (<2.5 m vs. >2.5 m), Cultivar (cv. Cogshall vs. cv. José), Harvest date (1 week between two harvests), as well as different fruit parts (Stem-end “SE” and peel surface “PE”) were studied. Bacterial and fungal community analyses demonstrated that these factors had an impact on both bacterial and fungal communities and the orchard (terroir), the cultivar, the harvest date, and fruit parts had the major impact on microbial communities.

Bacterial communities were significantly different between samples from the two mango cultivars. On the other hand, the differences in the fungal communities composition were highly significant between the two geographical locations, and significant different fungal populations were detected in the two different mango surface zones (Stem End vs. Cheek). Terroir can be defined by a set of agricultural parcels, which must be located in the same region, correspond to the same type of soil, both geologically and geographically, have identical climatic conditions and managed using the same technical cultural itineraries. Constancias and colleagues previously studied the distribution of soil microbial communities across an agricultural landscape and demonstrated that microbial biomass and bacterial richness distributions were mainly explained by soil pH and texture whereas bacterial evenness distribution was mainly related to land management (Constancias et al., 2015b). The “terroir” effect between St-Gilles and St-Pierre is probably a mixed between rootstock, soil and climate differences. Otherwise, all trees from a single plot have about the same age and a similar physiology. The type of soil, potential evapotranspiration, global radiation, precipitation, and temperature are factors related to the geographical location, which may have an impact on fruit quality, and therefore may have contributed to the observed differences. Reunion Island is made up of two large strato-volcanoes, the two sites chosen for the studies belong to different volcanoes, and therefore are composed of slightly different types of soil. Besides, Reunion Island is known for its multiple climatic micro-zones. A high level of humidity characterizes St-Pierre region, which is related to rainfall, as compared to St-Gilles region (Figure 1). Soil type was shown to harbor a wide variety of microbial communities and is the primary source of variability at the fruit surface (Ottesen et al., 2013; Constancias et al., 2014; Zarraonaindia et al., 2015; Liu et al., 2016). Distinct groupings of microbial communities were associated with different tomato plants, and a gradient of similarity correlated to the distance of a given plant part to the soil (Ottesen et al., 2013). Bacterial genera shared between grapevine cultivars and the vineyard soil can reach 60% of the microbiota (Mezzasalma et al., 2018). Geographical differentiation between bacterial and fungal communities associated with fruits is related to cultivars and species, according to Vepštaitė-Monstavičė et al. (2018). Even the “rootstocks” and grafted scion influences the fruit microbiota (Liu et al., 2018), which can select and affect bacterial richness and evenness (Marasco et al., 2018). Studies on the microbial ecology of the rootstock indicated that the soil is the primary source of microbial taxa found in the different organs of the plant (Hartmann et al., 2009; Martins et al., 2013; Reinhold-Hurek et al., 2015; Zarraonaindia et al., 2015).

There are two types of soil microbial transmission to the fruit surface: vertical transmission through the roots and the stomata, and horizontal transfer by wind carrying microbes from the environment to the fruit surface. The colonization and distribution of microorganisms depend on plant species and may be affected by growth and ripening stage (Leff and Fierer, 2013; Pinto et al., 2015). The ripening stage includes the morphology, physiology, and anatomy of the fruit, which plays a role in the selection and regulate the microbial diversity on the fruit surface. Differences in microbiome between cultivars were already showed in previous studies (Bokulich et al., 2014; Mezzasalma et al., 2018). These differences can be interpreted by physiological and morphological differences between the two mango cultivars used in this study, as it is known that the peel thickness are cultivar-specific (Konarska, 2012). Each cultivar produce fruit that are different in terms of size, skin thickness, and biochemical composition. Various studies showed that polyphenols content could influence microbial diversity (Daglia, 2012; Cardona et al., 2013; Duda-Chodak et al., 2015; Wang et al., 2016), suggesting that the secretion of phenolic compounds by mango peel may have an impact on their epiphytic microbiota. In general, both biotic and abiotic factors play critical roles for microbial community composition, richness, and diversity. It was shown that soil harbor a wide variety of microbial communities and is the primary source of variability at the fruit surface (Ottesen et al., 2013; Constancias et al., 2014; Zarraonaindia et al., 2015). The most dominant species we found in all samples belonging to Proteobacteria, Actinobacteria, Bacteroidetes, Firmicutes, and Acidobacteria phyla. Unsurprisingly, these bacterial phyla are ubiquitous in the soil and usually found on the surface of fruits and vegetables (Leff and Fierer, 2013; Constancias et al., 2015a).

Our results also show a significant difference in microbial communities, particularly the fungal population in two different part of cv. Cogshall fruits (SE and PE). Different parts of fruit have already reported in previous studies showing specific microbial communities (Abdelfattah et al., 2016a). On the one hand, a microenvironment different from the rest of the fruit may exists in the peduncle, especially in terms of humidity, availability of the nutrient. On the other hand, the morphology around the peduncle allows contamination and colonization. Comparison of stem-end microbial communities (SE) of cv. Cogshall with cv. Shelly showed that despite the fact that they share similar fungal families (i.e., *Pleosporaceae*, *Botryosphaeriaceae*, *Sporidiobolaceae*, *Quambalariaceae*, and *Dothioraceae*) they harbored different fungal composition. A few large families of fungi exclusively detected in the cv. Shelly stem-end, including *Davidiellaceae*, *Trichocomaceae*, *Metschnikowiaceae*, *Hypocreaceae*, and *Sclerotiniaceae*, suggested a link between microbiota and stem end disease (Diskin et al., 2017). This hypothesis was reinforced by the fact that the incidence of stem rot disease has a very low occurrence when compared to anthracnose in Reunion Island (unpublished data).

Fungal species and genera such as Alternaria, Bipolaris (Taba et al., 2007), Stomiopeltis (Ajitomi et al., 2017), Nigrospora (Alam et al., 2017, 2020), Passalora (i.e., Passalora fulva, syn. Cladosporium fulvum) (Altin, 2016), Stagonospora, Ascochyta (Milgroom and Peever, 2003), Diaporthe, Gibberella (Guenther and Trail, 2005), Mycosphaerella, Zasmidium, Sterigmatomyces, Helicoma, Puccinia, Exobasidium, Lasiodiplodia, Exserohilum, Curvularia (Oeurn et al., 2015; Hafiz et al., 2019), Phytophthora, Peniophora (Taylor, 1969), and Neofusicoccum (Hara et al., 2016) are commonly associated with plant disease and are most likely opportunists, which develop on stressed or dying fruit and leaf tissues (Om et al., 1999; Galsurker et al., 2018). Alternaria alternata were associated with mango stem-end root and C. gloeosporioides with mango anthracnose, resulting in serious post-harvest losses (Prusky et al., 2013; Diskin et al., 2017). Anthracnose is the most common disease in Reunion Island when compared to other microbial mediated plant diseases affecting mango (unpublished data). Lasiodiplodia sp. were also reported to be associated with dieback and stem-end rot of mango in the semi-arid region of Latin America (Marques et al., 2013; Rodríguez-Gálvez et al., 2017). Species of Stomiopeltis were reported causing flyspeck of mango (Ajitomi et al., 2017). Aspergillus and Botryosphaeria reported causing soft rot and dry rot on several fruit, including mango, pomegranates (Al-Najada and Al-Suabeyl, 2014; Li et al., 2020). Even though potential plant pathogens were identified in our metabarcoding 16S and ITS datasets, complementary experiments would be required to link the presence or density of potential pathogens and the occurrence of plant disease and additional measurements would be actually required to precisely quantify their density in the mango carposphere such as qPCR quantification or isolation. Nevertheless, the quality of the applied metabarcoding approach from swab sampling, DNA extraction and bioinformatic analyses seems to be sensitive enough to detect potential plant pathogens on obviously unaffected fruits.

In an orchard, the localization of the tree in the plot, the orientation, and position of the fruit on the tree can affect the diversity and composition of fruit microbiome. Trees closely located to a road can easily be exposed passively transported microbiota from unrelated environments (i.e., dust, air-polluting particles, aerosols). Thus, border trees may exchange the microbiota with trees of different cultivars, species, or intercropping (i.e., trap culture, repellant intercropping). The present study showed high bacterial diversity and richness on fruits harvested at the edge of the plot when compared to the center, but no significant difference in fungal richness and diversity were observed in terms of tree position. Previous studies on mango fruits have reported that the position on the canopy and exposure to the sunlight affects the accumulation of water, structural and non-structural dry matter in the fruit during its development (Léchaudel and Joas, 2007; Joas et al., 2013; Sivankalyani et al., 2016) reported that the effect of sun exposure on the accumulation of anthocyanin and flavonoids in the peel surface is related to resistance to mango anthracnose. A gradient of the composition of bacterial and fungal communities has been identified previously on a different part of the plant (Ottesen et al., 2013; Abdelfattah et al., 2016b; Trivedi et al., 2020). Our results did not confirm such pattern when considering orientation (East, North, and South) and position of the fruit on each tree (Height: <2.5 m” vs. >2.5 m”) on both bacterial and fungal diversity. The harvest date is strongly linked to climatic condition and mostly rainfall in the context of this study. High rainfall between the two sampling dates can leach microorganisms that are not adequately attached to the fruit surface. On the other hand, fungal communities can benefits from higher humidity. Fruit surface microbial adhesion is a critical step in biofilm formation, and therefore, promotes resistance to washing by rainwater and splashing raindrops. The waxy surface of the fruit peel presents an obstacle for bacterial adhesion, at least for many bacterial species (Reisberg et al., 2013). The difference in topology, morphology, and biochemical characteristics between the parts of the fruit may affect the fungal and bacterial species colonization and development.

## Conclusion

Microbial communities of the mango fruit surface are likely to be influenced by different factors at different scales. In this study, we inventoried and described the both bacterial and fungal communities associated with the mango carposphere, according to various orchard-linked features including the terroir (plot), the position of the tree in the orchard, the position the fruit on the tree, the orientation and the fruit parts and their impacts on associated microbial communities (diversity and composition). Our data also showed that, despite the presence of the factors influencing the microbiota of mango, different cultivars shared a common microbiota (core-microbiome) regardless of the geographical origin of the fruits. We noticed that geographical location had the most significant influence on the structures of both fungal and bacterial communities associated with cv. Cogshall surface. In general, we conclude that the cultivar showed less impact on fungal communities compared to the geographical location. This study contributes to a more in-depth knowledge of mango fruit microbiota which could lead to fruit microbiota-based orchard management, future biological control strategies, and processing and could be used for the development of a strategy based on mango microbiome manipulation to prevent post-harvest decay.

## Data Availability Statement

The datasets presented in this study can be found in online repositories. The names of the repository/repositories and accession number(s) can be found below: https://www.ncbi.nlm.nih.gov/, PRJNA682301.

## Author Contributions

AT designed and performed the experiments, analyzed the data, and prepared the original draft. AT, RR, J-CM, and FC contributed to the molecular biology and sequencing. FC performed bioinformatic analyses and data curation. J-CM and FC supervised the research, designed the experiments, and co-wrote the manuscript. VB, SL, and DP provided funding acquisition and project administration. VB co-wrote the manuscript. All authors approved the manuscript.

## Conflict of Interest

The authors declare that the research was conducted in the absence of any commercial or financial relationships that could be construed as a potential conflict of interest.

## Footnotes

1 https://itol.embl.de

## Supplementary Material

Supplementary Data 1 Table 1: Richness estimators and alpha diversity results of metagenomics surveys conducted with cv. Cogshall and cv. José samples originated from St-Gilles and St-Pierre. Shannon index (evenness of the species abundance distribution); Chao 1 index [richness + (estimated) number of unobserved species]; Observed species (number of observed species), and InvSimpson index (inverse probability that two sequences randomly sampled come from the same species). The results are presented as means ± SD. H1 and H2 represent two harvest campaigns of cv. Cogshall in St-Gilles: H3 and H4 in St-Pierre. JH1 (José harvested in St-Gilles) and JH2 (José harvested in St-Pierre) Stem-end (SE); Peel surface (PE).

Supplementary Data 2 Table 2: Kruskal–Wallis tests on α-diversity metrics: we used Kruskal–Wallis tests to assess whether alpha diversity was significantly different between categories. We used four different measurements of alpha diversity (Observed number of ASVs, Chao1, Shannon, and Inverse Simpson indices). The asterisk represents the level of significance of differences between categories. The stars denote significance *p*-value: (ns) indicate *P* > 0.05; (^∗^) *P* ≤ 0.05; (^∗∗^) *P* ≤ 0.01; (^∗∗∗^) *P* ≤ 0.001.

Supplementary Data 3 Table 3: Statistical analysis of category effects on bacterial and fungal diversity patterns conducted with, ANOSIM (analysis of similarity); PERMANOVA (Permutational multivariate analysis of variance); and MRPP (multi-response permutation procedures) based on Sørensen, Jaccard, and Bray-Curtis distance metrics. *P*-value (i.e., significance levels), *R*-value (i.e., the strength of the factors on the samples). *R*-value close to 1 indicates high separation between categories of the factor (e.g., St-Gilles vs. St-Pierre samples), while *R*-value close to 0 indicate no separation categories levels of the factor. Pseudo-F (PERMANOVA pseudo-F-statistic), *R*^2^ is about explanatory power; A (i.e., chance-corrected within-group agreement).

Supplementary Data 4 Differential abundance analysis (DESeq2) of fungal ASVs applied to different factors, including plot, cultivar, harvest date, and fruit parts.

Supplementary Data 5 The relative abundance of fungal (ITS) composition in terroirs, cultivars and at the fruit scale. Average (Mean) of relative abundance (%) at phylum, order, family, genus and species level were calculated and presented with standard deviation (SD) in samples according to regions, cultivars, and fruit parts.

Supplementary Data 6 The relative abundance of bacterial (16S) composition in terroirs, cultivars and at the fruit scale. Average (Mean) of relative abundance (%) at phylum, class, order, family, genus and species level were calculated and presented with standard deviation (SD) in samples according to regions, cultivars, and fruit parts.

Supplementary Data 7 Differential abundance analysis (DESeq2) of bacterial ASVs applied to different factors, including plot, cultivar, fruit parts, harvest date, tree position, height, and orientation.

Supplementary Data 8 Overview of the main factors. (S8-1) Boxplots of comparison of diversity Shannon between samples from different plots, cultivars, harvest dates and fruit parts, Fungal **(A)** and Bacterial ASVs **(B)**. (S8-2) Non-metric multidimensional scaling (NMDS) plot based on the distance matrix of Bray–Curtis distance metric of the microbial community between all samples, **(A)** Fungal and **(B)** Bacterial ASVs. (S8-3) Global UpSetR plot of unique and shared bacterial **(A)** and fungal ASV **(B)** among all samples harvested in St-Gilles and St-Pierre.
