# Supplementary material for: Terroir Is the Main Driver of the Epiphytic Bacterial and Fungal Communities of Mango Carposphere in Reunion Island
Source: Front Microbiol. 2021 Jan 20;11:619226. doi: 10.3389/fmicb.2020.619226 (PMC7874004; doi:10.3389/fmicb.2020.619226)
Supplement: Supplementary Data 8 — Overview of the main factors. (S8-1) Boxplots of comparison of diversity Shannon between samples from different plots, cultivars, harvest dates and fruit parts, Fungal (A) and Bacterial ASVs (B). (S8-2) Non-metric multidimensional scaling (NMDS) plot based on the distance matrix of Bray–Curtis distance metric of the microbial community between all samples, (A) Fungal and (B) Bacterial ASVs. (S8-3) Global UpSetR plot of unique and shared bacterial (A) and fungal ASV (B) among all samples harvested in St-Gilles and St-Pierre. [file Data_Sheet_1.PDF]

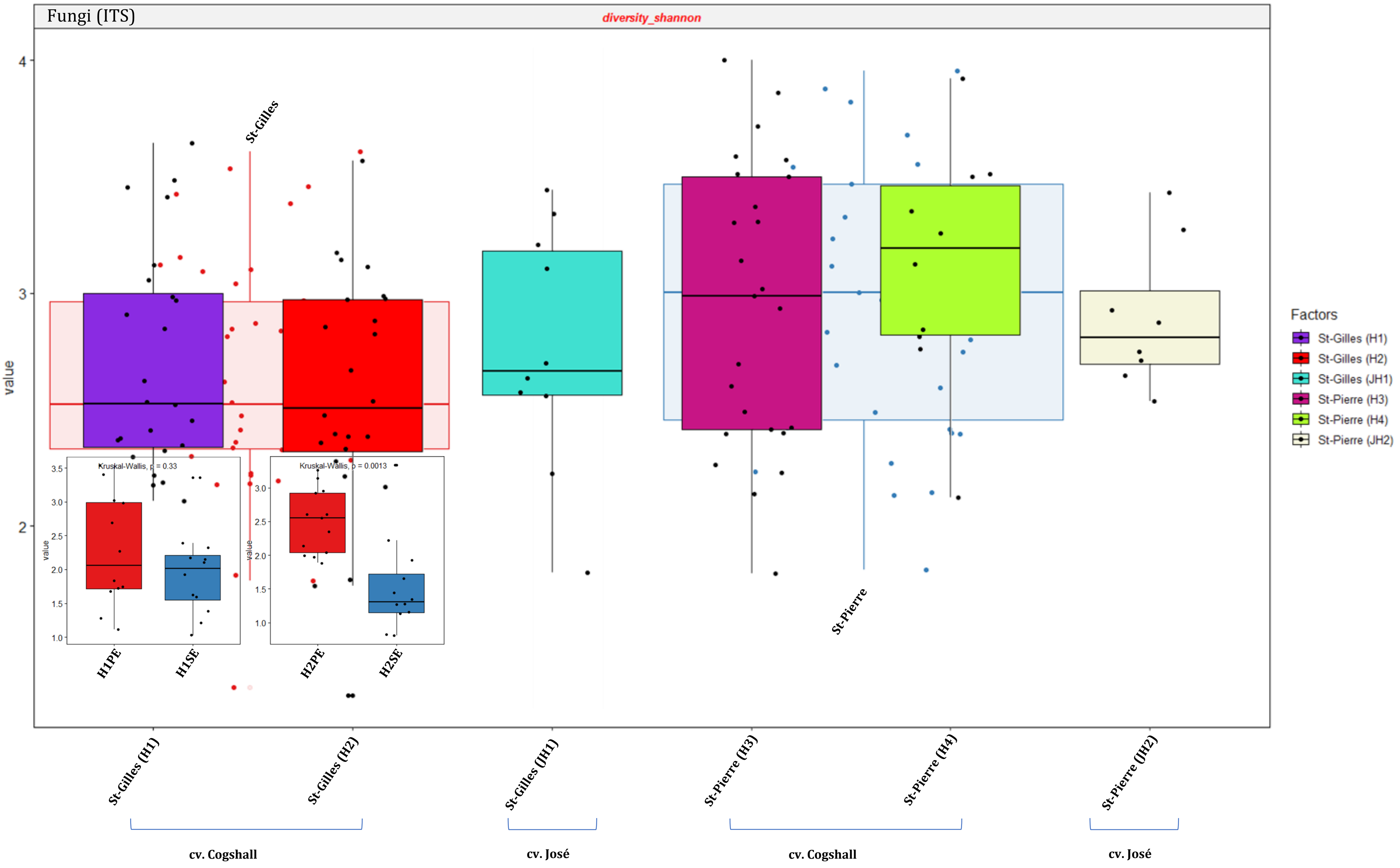

Fungi (ITS)
diversity_shannon
value
St-Gilles
St-Pierre
Kruskal-Wallis, p = 0.33
Kruskal-Wallis, p = 0.0013
H1PE
H1SE
H2PE
H2SE
St-Gilles (H1)
St-Gilles (H2)
St-Gilles (JH1)
St-Pierre (H3)
St-Pierre (H4)
St-Pierre (JH2)
cv. Cogshall
cv. José
cv. Cogshall
cv. José
Factors
St-Gilles (H1)
St-Gilles (H2)
St-Gilles (JH1)
St-Pierre (H3)
St-Pierre (H4)
St-Pierre (JH2)

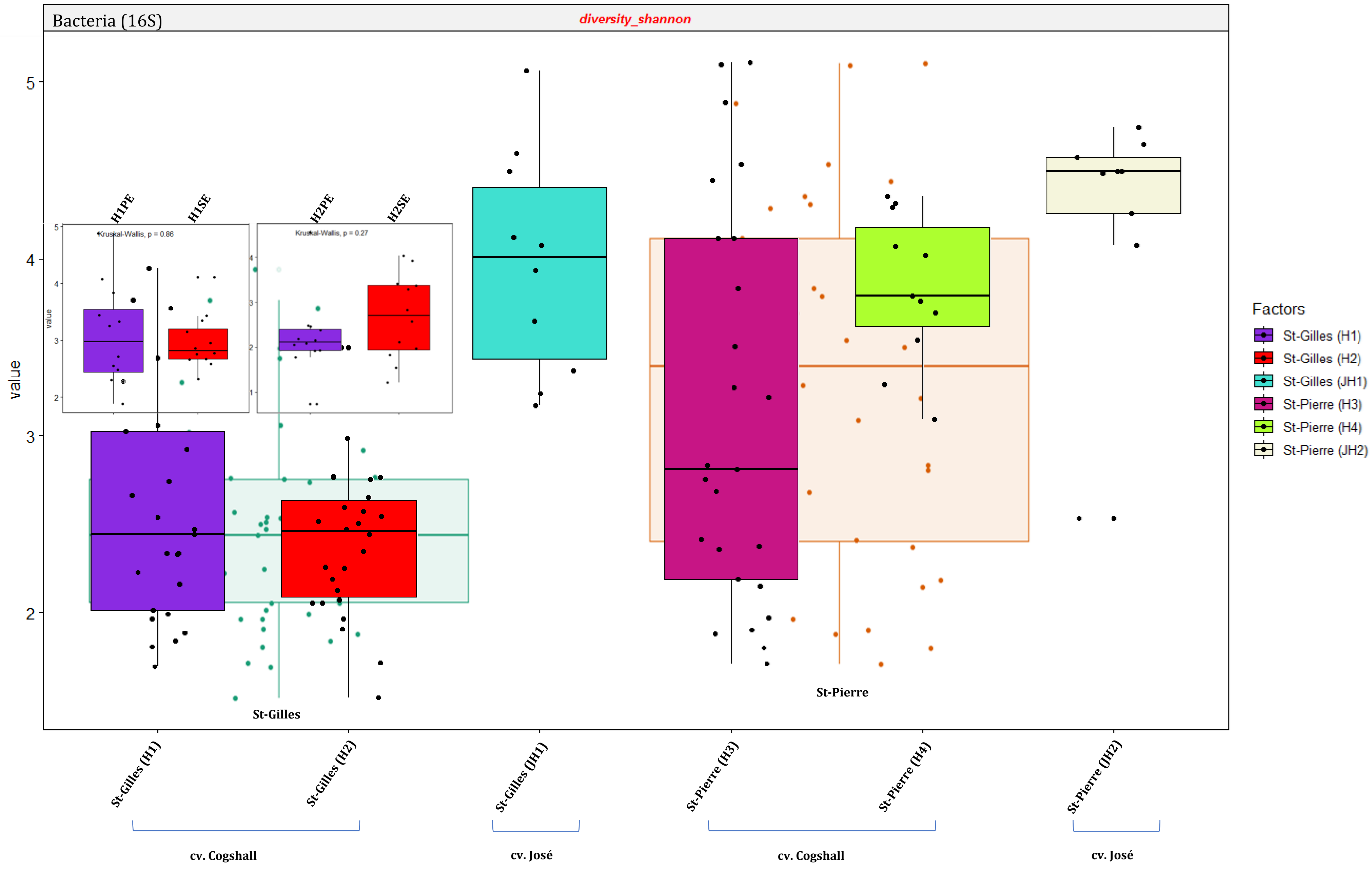

Bacteria (16S)
diversity_shannon
value
H1PE
H1SE
H2PE
H2SE
Kruskal-Wallis, p = 0.86
Kruskal-Wallis, p = 0.27
St-Gilles
St-Pierre
St-Gilles (H1)
St-Gilles (H2)
St-Gilles (JH1)
St-Pierre (H3)
St-Pierre (H4)
St-Pierre (JH2)
cv. Cogshall
cv. José
cv. Cogshall
cv. José
Factors
St-Gilles (H1)
St-Gilles (H2)
St-Gilles (JH1)
St-Pierre (H3)
St-Pierre (H4)
St-Pierre (JH2)

Fungi (ITS)

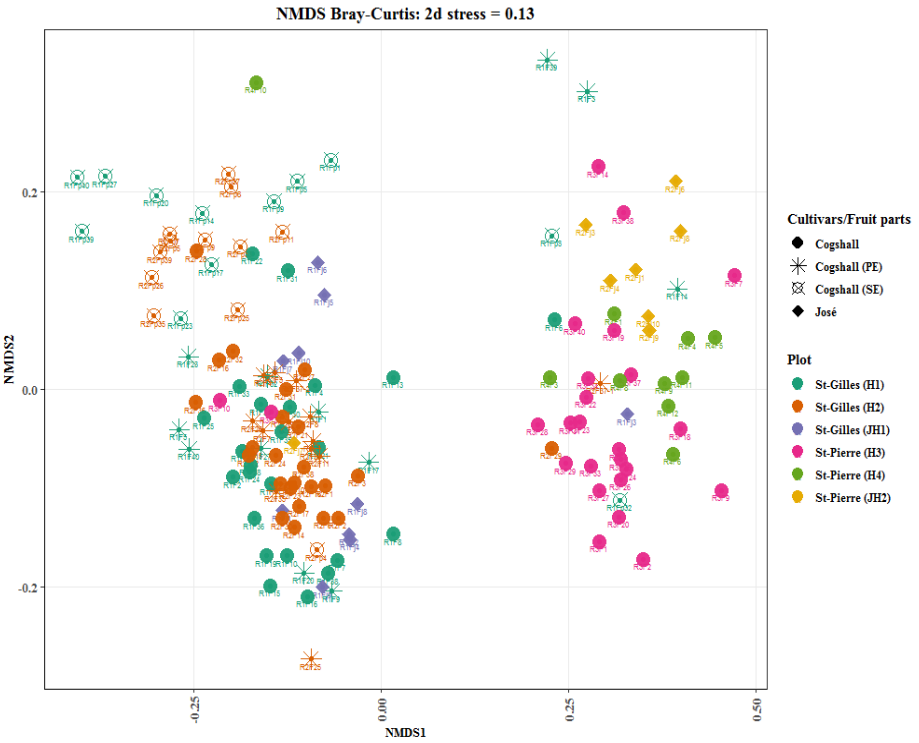

Permanova :
Plot : $R^2 = 0.3181$; $P = 0.001$ ***
Cultivars/Fruit parts : $R^2 = 0.02880$; $P = 0.004$ **

Bacteria (16S)

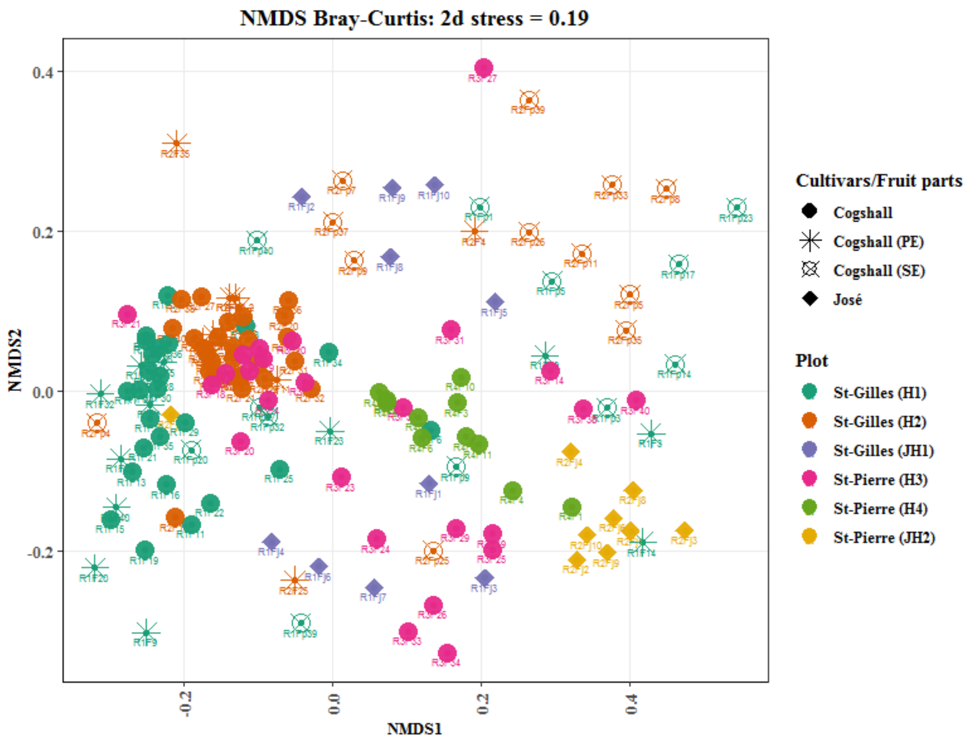

Permanova :
Plot : $R^2 = 0.1649$; $P = 0.001$ ***
Cultivars/Fruit parts : $R^2 = 0.10735$; $P = 0.001$ ***

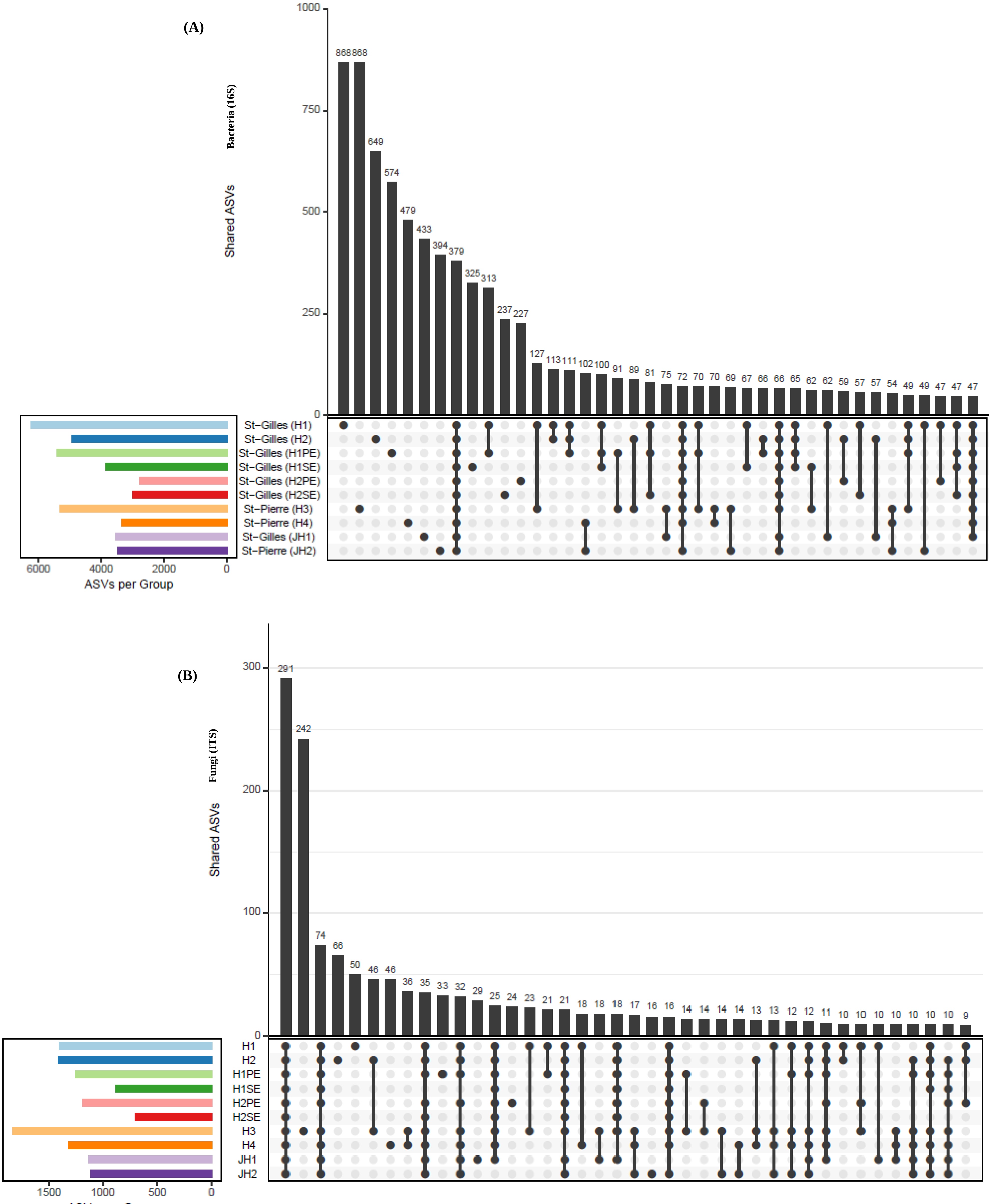
(A)
Bacteria (16S)
Shared ASVs
1000
750
500
250
0
868 868 649 574 479 433 394 379 325 313 237 227 127 113 111 102 100 91 89 81 75 72 70 70 69 67 66 66 65 62 62 59 57 57 54 49 49 47 47 47
St-Gilles (H1)
St-Gilles (H2)
St-Gilles (H1PE)
St-Gilles (H1SE)
St-Gilles (H2PE)
St-Gilles (H2SE)
St-Pierre (H3)
St-Pierre (H4)
St-Gilles (JH1)
St-Pierre (JH2)
6000
4000
2000
0
ASVs per Group
(B)
Fungi (ITS)
Shared ASVs
300
200
100
0
291 242 74 66 50 46 46 36 35 33 32 29 25 24 23 21 21 18 18 18 17 16 16 14 14 14 14 13 13 12 12 11 10 10 10 10 10 10 10 9
H1
H2
H1PE
H1SE
H2PE
H2SE
H3
H4
JH1
JH2
1500
1000
500
0
